# Supplementary material for: Occurrence of concurrent infections with multiple serotypes of dengue viruses during 2013–2015 in northern Kerala, India
Source: PeerJ. 2017 Mar 14;5:e2970. doi: 10.7717/peerj.2970 (PMC5354076; doi:10.7717/peerj.2970)
Supplement: Supplemental Information 1 [file peerj-05-2970-s001.docx]

GenBank accession no. KX031992

https://www.ncbi.nlm.nih.gov/nuccore/KX031992

>KX031992.1 Dengue virus polyprotein gene, partial cds

CGCGAGAAACCGTGTGTCAACTGGATCACAGTTGGCGAAGAGATTCTCAAAAGGATTGCTGAACGGCCAG

GGACCAATGAAATTGGTTATGGCGTTCATAGCCTTCCTTAGATTTCTGGCCATTCCACCAACAGCAGGAG

TTTTGGCCAGATGGGGGACCTTCAAGAAGTCGGGGGCCATTAAGGTCCTGAAAGGCTTCAAAAAGGAGAT

TTCAAACATGCTGAGCATAATCAACAAACGGAAAAAGACATCGCTCTGTCTCATGATGATATTACCAGCA

GCACTTGCTTTCCACTTGACTTCACGAGATGGAGAGCCGCGCATGATTGTGGGGAAAAATGAAAGAGGAA

AATCCCTACTTTTTAAGACAGCCTCTGGAATCAACATGTGCACACTCATAGCCATGGACTTGGGAGAGAT

GTGTGATGACACGGTCACTTACAAATGCCCCCACATTACCGAAGTGGAACCTGAAGACATTGACTGTTTG

GTGCAAA

GenBank accession no. KJ954284

https://www.ncbi.nlm.nih.gov/nuccore/KJ954284

>KJ954284.1 Dengue virus strain CUKKEL201308001 polyprotein gene, partial cds

TCAATATGCTGAAACGCGCGAGAAACCGCGTGTCAACTGGTTCACAGTTGGCGAAGAGATTCTCAAAAGG

ATTGCTTTCAGGCCAAGGACCCATGAAATTGGTGATGGCTTTTATAGCATTCCTAAGATTTCTAGCCATA

CCCCCAACAGCAGGAATTTTGGCTAGATGGAGCTCATTCAAGAAGAATGGAGCGATCAAAGTGTTACGGG

GTTTCAAAAAAGAGATCTCAAGCATGTTGAACATAATGAACAGGAGGAAAAGATCCGTAACCATGCTCCT

CATGCTGCTGCCCACAGCCCTGGCGTTCCATTTGACCACACGAGGGGGAGAGCCACATATGATAGTCAGC

AAGCAGGAAAGAGGAAAGTCACTCTTGTTTAAGACCTCGGCAGGCATCAACATGTGTACCCTCATTGCAA

TGG

GenBank accession no. KM042094

<https://www.ncbi.nlm.nih.gov/nuccore/KM042094>

>KM042094.1 Dengue virus strain CUK/KEL2013/002 nonfunctional polyprotein gene, partial sequence

TCAATATGCTGAAACGCGCGAGAAACCGTGTGTCAACTGGATCACAGTTGGCGAAGAGATTCTCAAAAGG

ATTGCTGAACGGCCAGGGACCAATGAAATTGGTCATGGCGTTCATAGCCTTCCTTAGATTTCTGGCCATT

CCACCAACAGCAGGAGTCTTGGCCAGATGGGGAACTTTCAAGAAGTCGGGGGCCATCAAGGTCCTGAAAG

GCTTCAAGAAGGAGATTCAAACATG
